# Addressing the elephant in the screening room: an item response theory analysis of the Prodromal Questionnaire for at-risk symptoms of psychosis

**DOI:** 10.47626/1516-4446-2024-3614

**Published:** 2025-01-22

**Authors:** Christophe Gauld, Pierre Fourneret, Ben Alderson-Day, Emma Palmer-Cooper, Clément Dondé

**Affiliations:** 1 Service de Psychopathologie du Développement, Hospices Civils de Lyon, Lyon, France; 2 Institut des Sciences Cognitives Marc Jeannerod, Unité Mixte de Recherche 5229, Centre National de la Recherche Scientifique & Université Claude Bernard Lyon 1, Lyon, France; 3 Department of Psychology, Durham University, Durham, UK; 4 School of Psychology, Centre for Innovation in Mental Health, University of Southampton, Southampton, UK; 5 Univ. Grenoble Alpes, F-38000 Grenoble, France; 6 INSERM, U1216, F-38000 Grenoble, France; 7 Psychiatry Department, CHU Grenoble Alpes, F-38000 Grenoble, France; 8 Psychiatry Department, Centre Hospitalier Alpes-Isère, F-38120 Saint-Egrève, France

**Keywords:** Clinical high-risk symptoms, discrimination, item response theory, early intervention

## Abstract

**Objective:**

Patients at risk of psychosis present a variety of symptoms, and identifying the most discriminative symptoms is essential for efficient detection and treatment.

**Methods:**

This cross-sectional online study analyzed individuals from the general population to better assess their risk of symptoms classified as clinical high risk (CHR) for psychosis. The 16-item Prodromal Questionnaire was applied as a self-report screening tool. Item response theory with a graded response model was used to assess the discrimination and difficulty of the questionnaire’s criteria.

**Results:**

The analysis included 936 participants (mean age: 21.5 years; 71.9% women). “*Déjà vu*” stood out for its high discriminative power, while the “voices or whispers” and “seeing things” items had greater precision than the other CHR-related symptoms. Conversely, the “smell or taste” and “changing faces” items were associated with the most severe cases.

**Conclusion:**

This study identified the most indicative CHR-related symptoms for accurate assessment of psychosis severity, which can be used to guide targeted preventative interventions.

## Introduction

Identifying individuals at risk of psychosis has become a central objective in modern clinical practice. The concept of clinical high risk (CHR) of psychosis, also referred to as attenuated psychosis syndrome in the DSM-5, serves as an inclusive clinical construct for identifying potentially prodromal manifestations of psychotic disorders.^1^,^2^ However, community screening approaches are associated with a significant false-positive rate, reaching approximately two-thirds of screened individuals.^3^ Recent research indicates that questionnaire items endorsed by a substantial percentage of the general population who do not seek help may capture more normative experience rather than symptomatology associated with psychosis risk.^4^ Notably, some participants reported distress but not psychotic-like experiences, which are more commonly assessed in the general population. The challenge is further compounded by the prevalence of numerous non-specific clinical manifestations. A recent analysis identified at least 68 distinct symptoms across validated CHR screening questionnaires.^5^ One solution for this heterogeneity would be to identify a more concise set of discriminating symptoms based on existing screening approaches. Thus, it seems particularly important to target items that best reflect the normative experiences and symptoms associated with psychosis risk. To this end, we employed item response theory (IRT) analysis on the widely used 16-item Prodromal Questionnaire (PQ-16), a tool “for screening potential at-risk mental states,”^6^,^7^ in large cohorts through population-based online screening.^3^ The objective of our study was to identify the most discriminative CHR symptoms measured in the PQ-16, without assumptions regarding formal diagnosis.

## Methods

### Participants

We recruited participants aged 18-35 from the non-help-seeking general population through Amazon’s Mechanical Turk and university mailing lists in France and the United Kingdom as part of the TONE-P study^8^ (see Methods in Supplementary Box S1 for details). To distinguish this sample from individuals who definitively meet CHR criteria, we analyzed schizotypal traits or psychosis, identified as the most relevant CHR-related symptoms on the PQ-16. Indeed, the PQ-16 is only for individuals at risk of developing psychosis; its factors reflect dimensions such as perceptual abnormalities/hallucinations and general symptoms associated with psychosis-risk.

### Questionnaire

The PQ-16 is a 16-item self-report screening questionnaire validated in English and French.^7^,^9^ Each item assesses anomalous psychotic experiences and associated distress on a 5-point Likert scale with five options (0 = none, 1 = any distress [symptom present without causing distress], 2 = mild distress, 3 = moderate distress, 4 = severe distress). More specifically, the scale first assesses the presence or absence of a symptom (0) and, if present, the level of distress associated with it (1 to 4). As a specific screening tool for CHR, endorsing six or more items considered is the threshold for a significant level of distress.^7^

### Item response theory

IRT analysis, specifically the graded response model, was used to evaluate the psychometric properties of each of the PQ-16’s items. IRT offers tools to more precisely assess symptom severity and differentiate between severity levels. It allows direct analysis of symptoms, rather than the construct itself. IRT framework considers two key parameters:

- Item “difficulty” reflects the severity level of the CHR-related symptom set (latent trait), with respondents having a 50% chance of endorsing the presence of any given symptom. It indicates how likely a respondent is to answer in a manner that corresponds to the underlying trait measured by the scale (without a unit of measurement). High difficulty response options (from 0 to 4) are more challenging to select due to factors such as complexity, ambiguity, or cognitive demand.
- Item “discrimination” refers to an item’s ability to differentiate between respondents with varying levels of the measured latent trait. It reflects how well an item can distinguish between individuals who are just above or below a specific point on the severity continuum. High discrimination indicates that the item effectively separates individuals with slightly different levels of the trait.

In our IRT model, the latent trait, representing the CHR-related symptom set, is a continuous variable determined by the relative difficulty and discrimination of each item. Option characteristic curves (analogous to item characteristic curves for ordinal data) are provided with their corresponding coefficients. We also reported factor loadings and communalities, which provide insight into the relationship between items and the latent trait. Finally, we evaluated model fit indices (infit and outfit) to ensure the IRT model appropriately reflects the data, items, and participant responses. Unidimensionality, a key assumption of the model, was verified through confirmatory factor analysis with established criteria: comparative fit and Tucker-Lewis indices ≥ 0.95, and root mean square error of approximation ≤ 0.06.^6^,^7^ All analyses and graphical visualizations were performed in R 4.3.1.

### Ethics statement

The study received ethical approval from the University Grenoble Alpes, France, Durham University, and Southampton University, United Kingdom. All procedures contributing to this study comply with the ethical standards of the relevant national and institutional care guidelines.

## Results

### Sample

A total of 936 participants were included in the analysis. Sociodemographic data and response patterns are reported in Table 1 and Supplementary Table S1, respectively. A total of 418 participants (44.42%) exceeded the PQ-16 cutoff (endorsing ≥ 6 items).

### Item response theory

**Figure 1:**
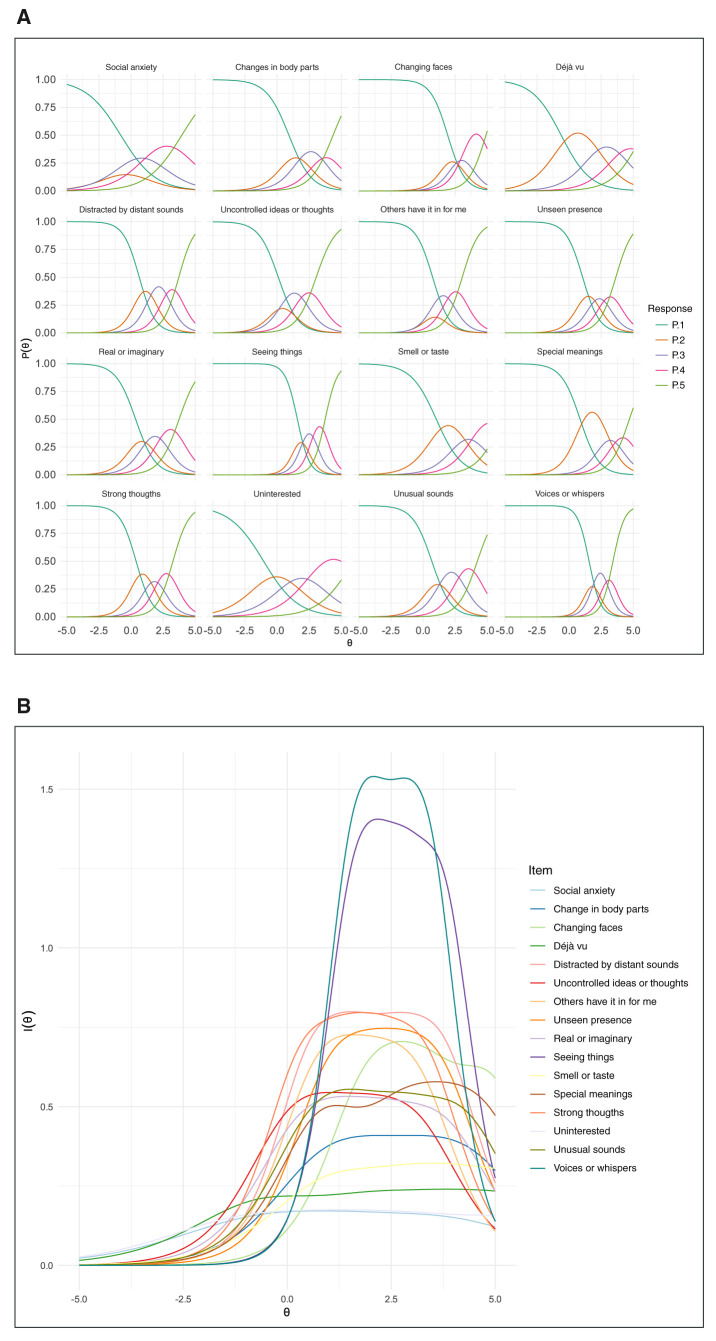
A) Option characteristic curves to visualize the discrimination and difficulty of each item of the Prodromal Questionnaire-16 for clinical high risk-related symptoms (n=936). The difficulty parameter is the point on the x axis where the probability of endorsing a criterion was 0.5; a curve to the right indicates criteria of greater difficulty relative to symptom severity level (*θ*). The discrimination parameter is the slope of the curve at that point, with steeper slopes indicating greater discrimination relative to symptom severity level (*θ*). B) The contribution of each item to total precision (or accuracy) of the PQ-16 regarding CHR-related symptoms (n=936). As the severity level of the CHR-related symptom set increased (to the right of *θ*), the probability of endorsing an item increased (except for Likert point 0, None) and then decreased as responses moved to the next higher Likert point (except for point 3, Severe). P.1 = none; P.2 = any; P.3 = mild; P.4 = moderate; P.5 = severe.

Item discrimination and difficulty parameters according to the graded response model are presented in Supplementary Table S2. The item “I often seem to live through events exactly as they happened before (“*déjà vu*”)” had the highest discrimination value (3.87), indicating a strong ability to differentiate between participants with varying levels of distress linked to CHR-related symptoms. This suggests a robust relationship between the *déjà vu* item and all other CHR-related symptoms (latent trait). *Déjà vu* also had high uniqueness (0.85) (see Supplementary Table S2), signifying that a substantial portion of its variance cannot be explained by the latent trait alone. This implies that while *déjà vu* effectively discriminates between individuals in the CHR-related symptom set, it also captures unique experiential aspects not shared by other PQ-16 symptoms. From a clinical standpoint, this suggests that *déjà vu* might tap into a distinct facet of latent trait, not fully captured by the questionnaire’s main factors, which highlights its importance as a unique indicator for clinical assessment. Consistent with this interpretation, steeper slopes in Figure 1 (panel A) indicate higher discrimination values. The probability of transitioning between response options on the scale became progressively more difficult (average relative difficulty coefficients, without units: 0.59, 1.70, 2.84, and 4.22). Notably, the items “I sometimes smell or taste things that other people can’t smell or taste” (3.57) (henceforth “Smell or taste”) and “When I look at a person, or look at myself in a mirror, I have seen the face change right before my eyes” (3.21) (henceforth “Changing faces”) were the most challenging response options.

For all items, the probability curve for the first response option (None) indicated that it was readily endorsed by participants who did not have a high level of distress from CHR-related symptoms (Figure 1, panel A) (i.e., up to an average level of latent trait severity). The subsequent three options (Any, Mild, and Moderate) showed good discrimination for most items, but shifted along the latent trait axis (reflecting difficulty) from left to right. The endorsement probability for these three options was relatively lower than for None or Severe. Finally, the endorsement probability for Severe was high for individuals with high level of symptom severity. However, for certain items (“I feel uninterested in the things I used to enjoy” [henceforth, “Uninterested”], “Smell or taste”, and “*Déjà vu*”), it became progressively more difficult to endorse Severe, as evidenced by a sharp shift to the right in the corresponding curve (Figure 1, panel A).

The items, “I have heard things other people can’t hear like voices of people whispering or talking” (henceforth, “Voices or whispers”) and “I have seen things that other people apparently can’t see” (henceforth, “Seeing things”) contributed most to precise measurement of distress level (Figure 1, panel B and Supplementary Figure S1). Conversely, “I get extremely anxious when meeting people for the first time” (henceforth, “Social anxiety”) and “Uninterested” had minimal influence on the precision of distress level measurement. Detailed information on factor loadings, communalities (the proportion of variance in an item explained by the latent trait), model fit indices, and applicability conditions can be found in Supplementary Tables S3 and S4, Supplementary Figures S1 and S2, and Supplementary Box S1. The items are described in full in Supplementary Table S5.

All criteria for model interpretability and applicability were met, with comparative fit and Tucker-Lewis indices > 0.95 (0.96) and root mean square error of approximation < 0.06 (0.046).

## Discussion

This study employed IRT analysis of the PQ-16 screening tool to identify the most discriminative CHR-related symptoms. We focused on both item difficulty (the symptom severity level at which endorsement is most probable) and discrimination (the ability to distinguish between individuals with different severity levels). These results demonstrate the model’s strong ability to differentiate between individuals across the severity spectrum. Specifically, as symptom severity increases, the probability of endorsing an item also increases. In other words, this IRT model reveals an item response gradient that reflects the latent trait of severity. Since selecting the most informative items for clinical use is a key challenge in this approach, this information could contribute to shorter, more accurate screening scales.

Among the assessed symptoms, *déjà vu* had the highest discrimination value, indicating a strong ability to differentiate between individuals with varying level of severity. This highlights this symptom’s key importance for improving specificity and, consequently, reducing the risk of overdiagnosis.^10^ The high discriminative power of *déjà vu* suggests potential alterations in source memory/monitoring processes, which aligns with empirical findings from CHR studies.^11^,^12^ These studies have shown that early auditory processing deficits, such as difficulty discriminating between pitches in non-verbal sounds, are linked to broader cognitive dysfunction and poorer functional outcomes in CHR patients.

Another interesting result was that extreme response options (None and Severe) strongly discriminated the presence and severity of CHR-related symptoms. Conversely, the discrimination of nuanced response options (any, mild, or moderate distress) was finer. For these intermediate options, endorsement became increasingly difficult (right shifting on the IRT scale). From a clinical perspective, the escalating difficulty of selecting higher response options could indicate that it is a challenge to discern the severity of CHR-related symptoms.^13^,^14^ Consequently, participants with an average severity level often endorse the lowest option across all items. This could hinder a clinician’s ability to detect CHR-related symptoms until they have become very severe. This finding aligns with prior IRT analyses of the PQ-16.^4^,^15^,^16^ However, unlike our study, which focused on identifying the most clinically relevant symptoms in the PQ-16, previous research has examined the entire PQ,^4^ a child-focused version,^16^ and a specific prenatal cohort.^15^

Our analysis identified the items “Smell or taste” and “Changing faces” as the most difficult response options, which suggests that these perceptual symptoms may be particularly indicative of the most severe cases of CHR-related symptoms. The association between these symptoms and multiple sensory modalities (visual, etc.) and their appearance in only the most extreme cases aligns with the concept that a higher disease load is associated with multiple sensory dysfunctions. The most informative items for estimating the severity of CHR-related symptoms (“Voices or whispers” and “Seeing things”) also pertain to perception, specifically auditory and visual alterations. These findings highlight how critically important it is for clinicians to assess such perceptual symptoms. Conversely, symptoms with low discriminatory power, such as “Uninterested” and “Social anxiety,” appear to be of limited utility in gauging the severity of CHR-related symptoms in clinical practice.

Several limitations should be considered. First, no clinical investigation was performed to determine whether participants scoring above the PQ-16 cutoff had a CHR for psychosis or a psychotic disorder. The PQ-16 cutoff score should also be interpreted with caution, since it was originally developed for a help-seeking sample^7^ and might not generalize to a broader population. Future studies should incorporate semi-structured CHR diagnosis interviews following screening for the specific symptoms identified in this study. Second, while our study is multicentric and involves a large number of participants, our sample may not be entirely representative and generalizable. This is primarily due to its high proportion of women. However, its sex distribution aligns with the literature on CHR symptoms.^1^,^2^ Third, even though the screened sample reflects CHR-related symptoms, potentially representing a clinical population, the participants’ status as students might reflect recruitment bias. Consequently, special caution is needed regarding generalizability claims. Finally, caution is also required when interpreting our results due to the model’s complexity and the large sample size required to estimate the numerous parameters involved. An even larger and more diverse sample would undoubtedly yield more informative results, particularly for a nuanced understanding of the most extreme response options.

Demonstrating that item difficulty and response options vary reinforces the idea that certain symptoms can better account for the severity of all CHR-related symptoms. Thus, the most representative symptoms can be screened in preventive interventions. In a brief executive summary, we present key insights that highlight the clinical relevance and applicability of these findings:

- This symptom-level study demonstrates the ability to differentiate individuals across severity levels; for instance, items like “Smell or taste” and “Changing faces” are particularly relevant for identifying severe cases.
- To accurately measure distress severity, clinicians should prioritize assessment of perceptual symptoms, such as “Voices or whispers” and “Seeing things.”
- Clinicians should pay special attention to *déjà vu* in assessments, since it had the highest discrimination value among PQ-16 items and captures distinct experiential aspects with high uniqueness.
- Extreme response options (None and Severe) showed strong discrimination for CHR symptoms, while nuanced options (Any, Mild, Moderate) provided finer gradation in discrimination and increased difficulty, which can increase the precision of clinical assessments.
- From a psychometric perspective, classifying items based on their degree of discrimination and difficulty provides a better the understanding of the PQ-16’s structure and could contribute to more precise screening tools for clinical use.
- More generally, identifying clinically relevant symptoms and individual distress levels could enhance early identification and intervention strategies.

## Disclosure

The authors report no conflicts of interest.

## Figures and Tables

**Table 1:** Demographic and symptom profiles of the study participants based on 16-item Prodromal Questionnaire scores (n=936)

Category	Value
Age (years), mean (SD)	21.5 (5.1)
Sex	
Male	263 (28.1)
Female	673 (71.9)
Population breakdown, n	
France	367
United Kingdom	569
Occupation distribution	
Students	764 (81.6)
Employed	119 (12.7)
Unemployed	53 (5.6)
PQ-16, mean (SD)	5.6 (3.5)

Data presented as n (%), unless otherwise specified.
