# Supplementary material for: Addressing the elephant in the screening room: an item response theory analysis of the Prodromal Questionnaire for at-risk symptoms of psychosis
Source: Braz J Psychiatry. 2025 Jan 22;47:e20243614. doi: 10.47626/1516-4446-2024-3614 (PMC12679692; doi:10.47626/1516-4446-2024-3614)
Supplement: Supplementary file 1 [file bjp-47-e20243614-suppl1.pdf]

http://doi.org/10.47626/1516-4446-2024-3614

Supplementary Box S1 Details on the methods, model, and results

Methods – Ethical statements. The Tone-P Study is a cross-sectional online study to investigate early auditory processing in non-help seekers screened for CHR. The Tone-P study was funded by Gorilla.sc. Participants provided informed consent online, after which a sociodemographic assessment and two screening questionnaires were administered: the 16-item Prodromal Questionnaire and the 9-item perceptual and cognitive aberrations questionnaire scale.

Methods – 16-item Prodromal Questionnaire. The items are summarized as follows in our study: (1) “Uninterested”, (2) “*Déjà vu*”, (3) “Smell or taste”, (4) “Unusual sounds”, (5) “Real or imaginary”, (6) “Changing faces”, (7) “Anxiety to meet”, (8) “Seeing things”, (9) “Strong thoughts”, (10) “Special meanings”, (11) “Uncontrolled ideas or thoughts”, (12) “Distracted by distant sounds”, (13) “Voices or whispers”, (14) “Others have it in for me”, (15) “Unseen presence”, (16) “Changes in body parts”. The total score resulted from the sum of the scores obtained for each of the 16 items. It was based on the Prodromal Questionnaire, a 92-item self-report measure (Loewy et al., 2005), which was based on the Schizotypal Personality Questionnaire (Raine, 1991). Tested in a general non-help-seeking population, the initial validation study revealed a three-factor structure (perceptual abnormalities/hallucinations, unusual thoughts, and negative symptoms). It was translated to French and validated in both adult and adolescent populations (Lejuste et al., 2021; Spillebout et al., 2023).

Methods – Participants. A total of 948 participants were included in the study and, after processing missing data, 936 participants were analyzed. The mean age was 21.5 years, with a median of 20.0 (SD, 5.1) years. Of these 936 participants, 367 were from France (39.2%) and 569 were from the UK (60.8%)(χ^2 = 43.59, p < 0.001), of whom 263 were men (28.1%) and 673 were women (71.9%)(χ^2 = 179.59, p < 0.001). Regarding employment status, 764 were students (81.6%), 119 were employed (12.7%), and 53 were unemployed (5.6%)(χ^2 = 989.21, p < 0.001). The French and UK groups significantly differed in age (t = 15.82, p < 0.001 [4.74 – 6.09]) but not sex (χ^2 ~ 0, p ~ 1) or occupation (χ^2 = 6, p = 0.20).

Methods – Item Response Theory. We conducted an item response theory analysis using a graded response model. The graded response model was suitable for analyzing ordinal data. We were particularly interested in the factor loadings (F1) and the communalities (h2). In the context of item response theory, “F1” should be > 0.5, indicating adequate factor loading. “F1” represents the saturation of the factor (how well the item represents the underlying factor), while “h2” (which is “F1” squared) represents its communality, indicating the variance the factor explains in an item.

Results. The analysis was conducted using full-information item factor analysis with a single factor. It successfully converged after 52 iterations of the expectation maximization algorithm, with a tolerance level of 1e-04. This analysis was performed in MIRT 1.41. The Broydon-Fletcher-Goldfarb-Shanno method was used to optimize the M-step, and Ramsay's method was used for expectation-maximization acceleration. Sixty-one rectangular quadrature points were used in the analysis, and the latent density was assumed to be Gaussian.

The information matrix was estimated using Oakes' method. The second-order test suggests that the model could be a possible local maximum, and the information matrix condition number was 574, indicating potential numerical issues in the inversion or calculation of the matrix.

The model’s log likelihood was -7339.27. In total, 64 parameters were estimated. Based on these estimates, the Akaike information criterion was calculated at 14806.54, while the Bayesian information criterion and sample-size adjusted Bayesian information criterion were 15116 and 14913, respectively.

Validity condition. To determine how well the model fit the data, rather than using a χ^2, we used M^2, which is specifically designed to assess the fit of the item response models. The M^2 was 414.9, suggesting a moderate fit between the model and the observed data. Confirmatory factor analysis (lavaan package 0.6.15 – optimization method with NLMINB) ended after 44 iterations. Both the Tucker-Lewis and the comparative fit index were 0.96, which is above the common threshold of 0.95, further confirming the model’s good fit to the data. The log-likelihood values for the user model and the unrestricted model resulted in an Akaike information criterion of 20952.556 and a Bayesian information criterion of 21107.488. The root mean square error of approximation was 0.0464, with a 90% CI (0.0406–0.0523), indicating a good model fit, and the probability of root mean square error of approximation being ≤ 0.05 was 0. The model had a relatively low standardized root mean square residual (0.060), indicating a good fit in terms of the standardized difference between observed and predicted correlations. Confirmatory factor analysis also showed that two factors fit the data best, which is consistent with a number of previous studies on the 16-item Prodromal Questionnaire (Howie et al., 2020). The χ^2 statistic of 292.21 with a p-value < 0.001 (1.82e-23) indicates that the two-factor model is a good fit for the data. The factors account for approximately 30.5% of the total variance, with the first factor explaining 16.8% and the second factor explaining 13.7%. This analysis shows the uniqueness of each item in the dataset, indicating how much variance in the item is not explained by the factors. The items with the highest uniqueness values were “Déjà vu” (0.849), “Special meanings” (0.854), and “Uninterested” (0.761), implying that they were less well explained by the two factors than the other items. On the other hand, the items with the lowest uniqueness (hence, most influenced by the factors) were “Voices or whispers” (0.470), “Uncontrolled ideas or thoughts” (0.510), and “Seeing things” (0.602). These items were primarily loaded on the first factor, except “Uncontrolled ideas or thoughts”, which was more associated with the second factor.

http://doi.org/10.47626/1516-4446-2024-3614

Supplementary Table S1 Frequency and percentage of Likert responses to each item of the Prodromal Questionnaire-16 (n=936)

Items	None	Any	Mild	Moderate	Severe
Uninterested	311 (33%)	305 (32%)	210 (22%)	101 (11%)	14 (1%)
Déjà vu	364 (39%)	411 (44%)	125 (13%)	32 (3%)	9 (1%)
Smell or taste	669 (71%)	205 (22%)	46 (5%)	18 (2%)	3 (0%)
Unusual sounds	630 (67%)	159 (17%)	110 (12%)	34 (4%)	8 (1%)
Real or imaginary	564 (60%)	186 (20%)	120 (13%)	55 (6%)	16 (2%)
Changing faces	844 (90%)	54 (6%)	26 (3%)	15 (2%)	2 (0%)
Social anxiety	353 (38%)	124 (13%)	233 (25%)	172 (18%)	59 (6%)
Seeing things	837 (89%)	56 (6%)	33 (4%)	12 (1%)	3 (0%)
Strong thoughts	578 (61%)	210 (22%)	89 (9%)	47 (5%)	17 (2%)
Special meanings	668 (71%)	227 (24%)	31 (3%)	11 (1%)	4 (0%)
Uncontrolled ideas or thoughts	495 (53%)	151 (16%)	180 (19%)	81 (9%)	34 (4%)
Distracted by distant sounds	636 (68%)	180 (19%)	91 (10%)	26 (3%)	8 (1%)
Voices or whispers	839 (89%)	51 (5%)	35 (4%)	11 (1%)	5 (1%)
Others have it in for me	655 (70%)	76 (8%)	124 (13%)	61 (6%)	25 (3%)
Unseen presence	720 (77%)	129 (14%)	57 (6%)	24 (3%)	11 (1%)
Changes in body parts	662 (70%)	156 (17%)	86 (9%)	25 (3%)	12 (1%)

http://doi.org/10.47626/1516-4446-2024-3614

Supplementary Table S2 Coefficients for discrimination and the three difficulty thresholds between the five modalities (Likert points) of each Prodromal Questionnaire-16 item for clinical high risk (n=936)

**Items**	**Discrimination (slope)**	**Difficulty threshold 1 (from “None” to “Any”)**	**Difficulty threshold 2 (from “Any” to “Mild”)**	**Difficulty threshold 3 (from “Mild” to “Moderate”)**	**Difficulty threshold 4 (from “Moderate” to “Severe”)**
Uninterested	0.78	0.75	-1.05	0.96	2.88
Déjà vu	0.85	0.89	-0.61	1.99	3.87
Social anxiety	0.95	1.02	1.03	**2.90**	**4.19**
Changes in body parts	1.24	1.34	0.68	1.58	2.84
Smell or taste	1.04	1.31	0.36	1.31	2.41
Unusual sounds	1.46	1.50	**1.92**	2.64	3.39
Uncontrolled ideas or thoughts	0.59	0.74	-0.80	0.00	1.64
Others have it in for me	**2.46**	2.13	1.58	2.14	2.87
Unseen presence	1.43	1.60	0.39	1.41	2.23
Changing faces	1.34	1.36	0.86	2.73	3.68
Real or imaginary	1.28	1.32	0.10	0.77	1.91
Special meanings	1.62	1.62	0.64	1.61	2.70
Distracted by distant sounds	2.34	**2.23**	1.56	2.06	2.81
Strong thoughts	1.45	1.53	0.74	1.12	2.03
Seeing things	1.61	1.54	1.06	1.94	2.77
Voices or whispers	1.13	1.15	0.92	2.00	3.29

The items are ordered according to the discrimination value. For example, in the last column, the coefficient indicates the difficulty of moving from the fourth to the fifth modality, as well as the ease of moving from the fifth to the fourth. Values in bold are the highest in each column.

http://doi.org/10.47626/1516-4446-2024-3614

Supplementary Figure S1 Contribution of the 16 items of the Prodromal Questionnaire-16 for clinical high risk (n=936) (equivalent to Figure 1, panel B, but including all the items)

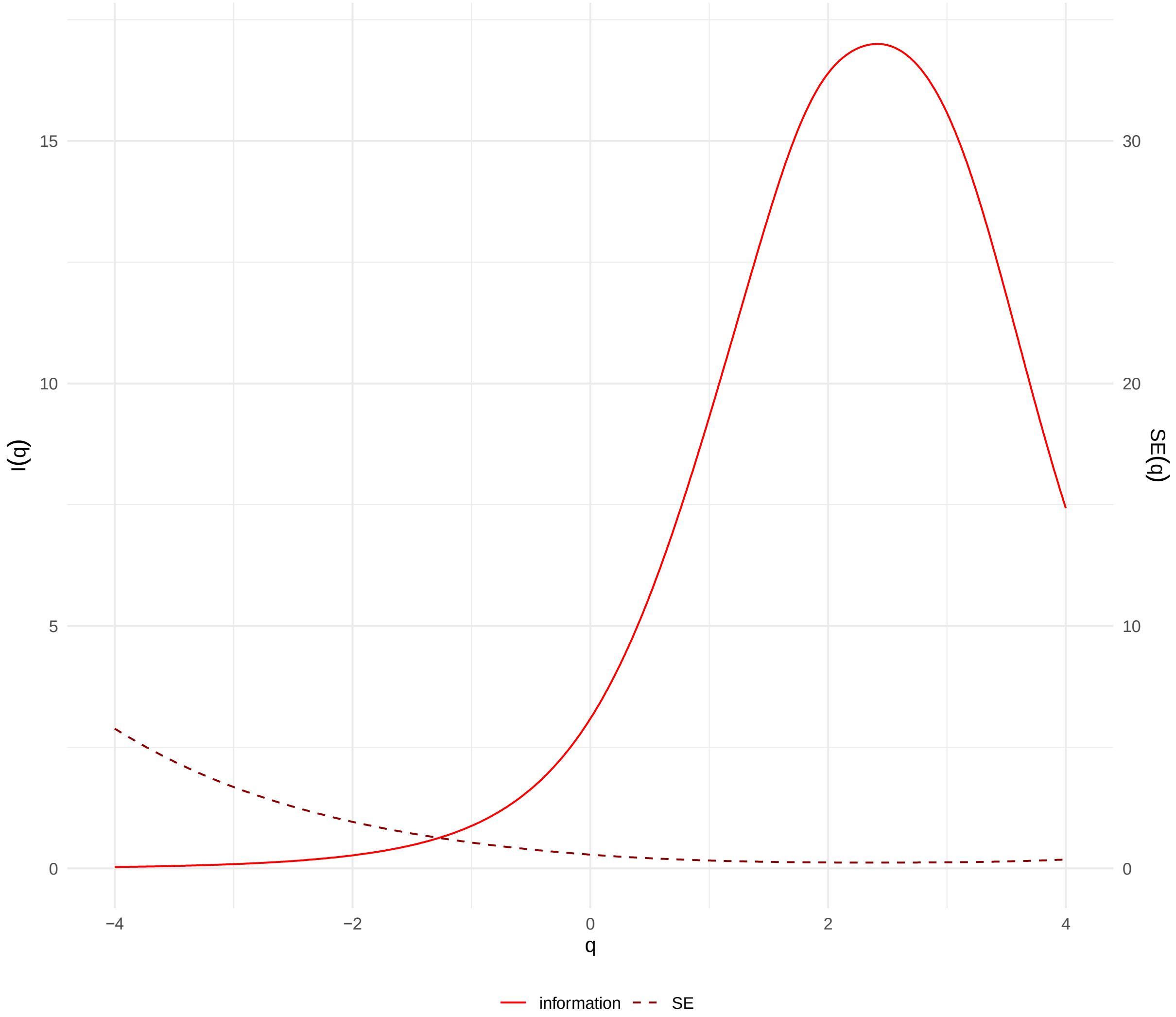

CHR = clinical high risk.
Regarding information precision, the items performed very well for a “high” CHR threshold and very poor for a “low” CHR threshold.

http://doi.org/10.47626/1516-4446-2024-3614

Supplementary Table S3 CHR factor loadings and communalities on the Prodromal Questionnaire-16 items (n=936)

**Items**	**CHR Factor loadings (F1)**	**Community (h2)†**
Uninterested	0.404	0.163
Déjà vu	0.462	0.213
Smell or taste	0.514	0.265
Unusual sounds	0.619	0.383
Real or imaginary	0.609	0.371
Changing faces	0.660	0.436
Anxiety to meet	0.398	0.159
Seeing things	0.781	0.610
Strong thoughts	0.685	0.469
Special meanings	0.625	0.390
Uncontrolled ideas or thoughts	0.613	0.376
Distracted by distant sounds	0.689	0.475
Voices or whispers	0.795	0.632
Others have it in for me	0.668	0.446
Unseen presence	0.671	0.450
Changes in body parts	0.558	0.312

CHR = clinical high risk.
† h2 represents the variance explained in an item by the latent trait.

http://doi.org/10.47626/1516-4446-2024-3614

Supplementary Table S4 The fit of Prodromal Questionnaire-16 items to the model (n=936)

Items	S_X2	Df (for S_X2)	RMSEA (for S_X2)	P-value (for S_X2)
Uninterested	94.885	71	0.019	**0.031***
Déjà vu	78.823	68	0.013	0.174
Smell or taste	72.934	50	0.022	**0.019***
Unusual sounds	76.230	69	0.011	0.257
Real or imaginary	124.851	73	0.027	**0.000***
Changing faces	55.005	46	0.014	0.170
Anxiety to meet	118.279	92	0.017	**0.034***
Seeing things	47.404	43	0.010	0.298
Strong thoughts	99.088	69	0.022	**0.010***
Special meanings	44.498	45	0.000	0.493
Uncontrolled ideas or thoughts	84.031	84	0.001	0.479
Distracted by distant sounds	89.327	62	0.022	**0.013***
Voices or whispers	50.223	43	0.013	0.209
Others have it in for me	103.945	75	0.020	**0.015***
Unseen presence	86.685	66	0.018	**0.045***
Changes in body parts	46.916	35	0.019	0.086

Df = degrees of freedom; RMSEA = root mean square error of approximation.
To study the fit of each item, we used Orlando & Thissen's (2000) S_X2 'and the corresponding root mean square error of approximation and p-values. This test must be insignificant to indicate a good fit. Significant p-values ($p < 0.05$, marked with "*") indicate less fit.

http://doi.org/10.47626/1516-4446-2024-3614

Supplementary Figure S2 Infit and outfit statistics showing the relation of the 16 Prodromal Questionnaire items to the model (n=936)

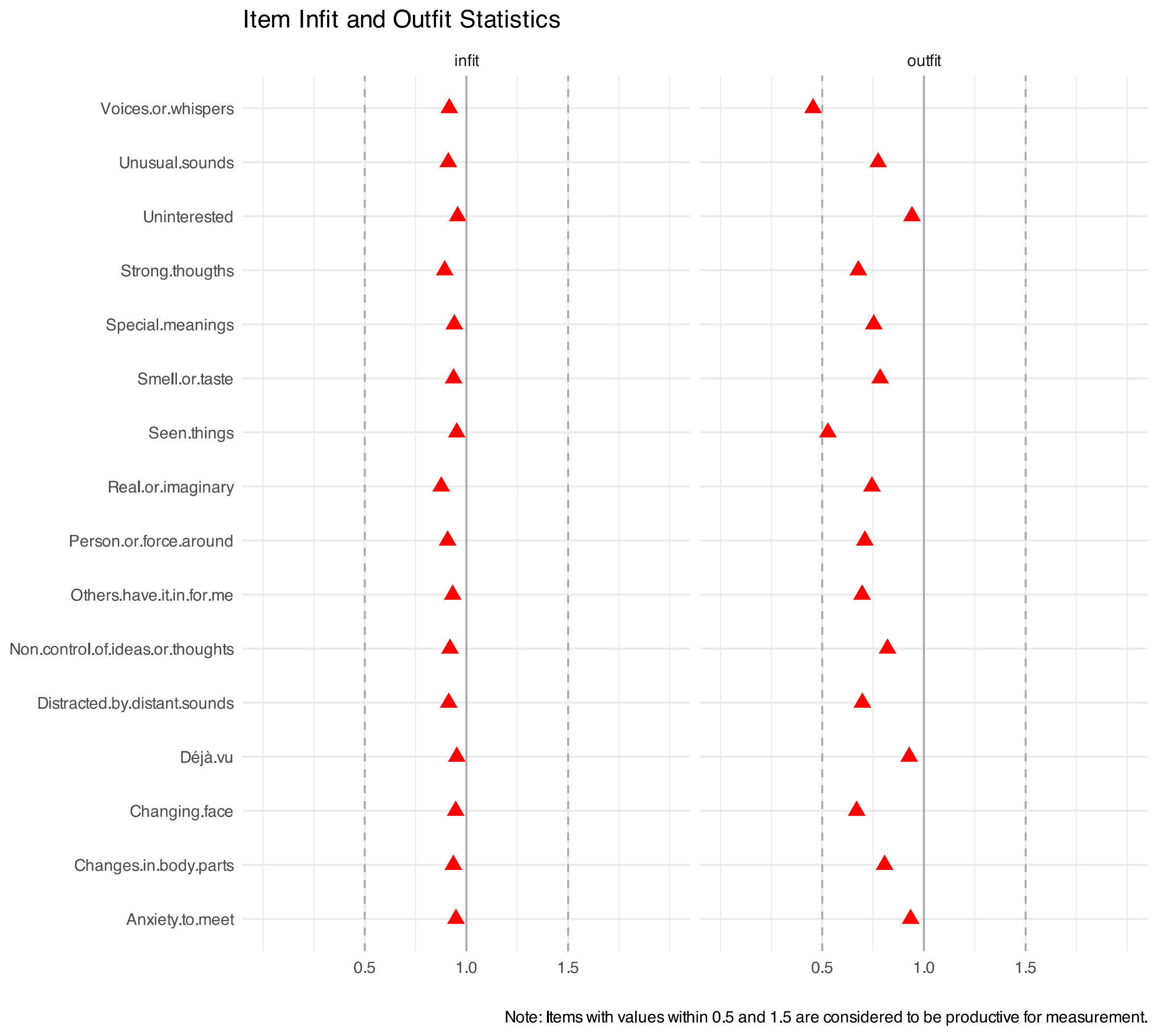

To not "degrade" (i.e., to be "productive" for measurement), non-standardized values must fall between 0.5 and 1.5.

http://doi.org/10.47626/1516-4446-2024-3614

Supplementary Table S5 Correspondence table

PQ-16 items	Item summary
I feel uninterested in the things I used to enjoy.	Uninterested
I often seem to live through events exactly as they happened before (déjà vu).	Déjà vu
I sometimes smell or taste things that other people can’t smell or taste.	Smell or taste
I often hear unusual sounds like banging, clicking, hissing, clapping or ringing in my ears.	Unusual sounds
I have been confused at times whether something I experienced was real or imaginary.	Real or imaginary
When I look at a person, or look at myself in a mirror, I have seen the face change right before my eyes.	Changing faces
I get extremely anxious when meeting people for the first time.	Anxiety to meet
I have seen things that other people apparently can't see.	Seeing things
My thoughts are sometimes so strong that I can almost hear them.	Strong thoughts
I sometimes see special meanings in advertisements, shop windows, or in the way things are arranged around me.	Special meanings
Sometimes I have felt that I’m not in control of my own ideas or thoughts.	Uncontrolled ideas or thoughts
Sometimes I feel suddenly distracted by distant sounds that I am not normally aware of.	Distracted by distant sounds
I have heard things other people can't hear like voices of people whispering or talking.	Voices or whispers
I often feel that others have it in for me.	Others have it in for me
I have had the sense that some person or force is around me, even though I could not see anyone.	Unseen presence
I feel that parts of my body have changed in some way, or that parts of my body are working differently than before.	Changes in body parts

PQ-16 = Prodromal Questionnaire-16.
